# *Marsdenia tenacissimae* extraction (MTE) inhibits the proliferation and induces the apoptosis of human acute T cell leukemia cells through inactivating PI3K/AKT/mTOR signaling pathway via PTEN enhancement

**DOI:** 10.18632/oncotarget.12654

**Published:** 2016-10-14

**Authors:** Ying Wang, Bingyu Chen, Zhen Wang, Wei Zhang, Ke Hao, Yu Chen, Kaiqiang Li, Tongtong Wang, Yiwei Xie, Zhihui Huang, Xiangmin Tong

**Affiliations:** ^1^ Key Laboratory of Laboratory Medicine, Ministry of Education, Wenzhou Medical University, Wenzhou, Zhejiang, 325035, China; ^2^ Institute of Neuroscience and Hypoxia Medicine, Wenzhou Medical University, Wenzhou, Zhejiang, 325035, China; ^3^ Department of Blood Transfusion, Zhejiang Provincial People's Hospital, Hangzhou, Zhejiang, 310014, China; ^4^ Clinical Research Institute, Zhejiang Provincial People's Hospital, Hangzhou, 310014, China

**Keywords:** Marsdenia tenacissimae extraction, T-cell acute lymphoblastic leukemia, proliferation, apoptosis, PTEN

## Abstract

*Marsdenia tenacissimae* extraction (MTE) as a traditional Chinese herb has long been used to treat some diseases such as tumors in China. However, the potential effectiveness of MTE in leukemia has not yet been fully understood, and the related molecular mechanism is still unknown. In the present study, we aimed to evaluate the effects of MTE on the proliferation and apoptosis of Jurkat cells (T-ALL lines) and lymphocytes from T-ALL (T-cell acute lymphoblastic leukemia) patients. Firstly, CCK8 assays and flow cytometry assays revealed that MTE dose-dependently reduced the proliferation of Jurkat cells by arresting cell cycle at S phase. Secondly, Annexin V-FITC/PI-stained flow cytometry and TUNEL staining assays showed that MTE promoted the apoptosis of Jurkat cells. Mechanistically, MTE enhanced PTEN (phosphatases and tensin homolog) level and inactivated PI3K/AKT/mTOR signaling pathway in Jurkat cells, which mediated the inhibition of cell proliferation by MTE and MTE-induced apoptosis. Finally, MTE significantly inhibited the proliferation and promoted the apoptosis of lymphocytes from T-ALL patients, compared with lymphocytes from healthy peoples. Taken together, these results reveal an unrecognized function of MTE in inhibiting the proliferation and inducing the apoptosis of T-ALL cells, and identify a pathway of PTEN/PI3K/AKT/mTOR for the effects of MTE on leukemia therapy.

## INTRODUCTION

Acute lymphoblastic leukemia (ALL) is an aggressive tumor of the hematopoietic system. Most of ALLs (85%) originate from the B-cell lineage, and the rest are from the T-cell lineage (15%) [1]. T-cell acute lymphoblastic leukemia (T-ALL) represents a malignant disorder, characterized by an uncontrolled accumulation of T-cell progenitors [1, 2]. Most human cases of T-ALL occur in young children between 2-5 years of age, but T-ALL can occur at any age [3]. Although the prognosis of pediatric T-ALL has recently improved due to intensified therapies, attaining more than 80% cure rates for children [4], there are many challenges including the early relapse of pediatric T-ALL, the poor prognosis of relapsed and primary chemo-resistant [1]. Hence, less toxic and more efficient and new therapeutic strategies are still required, especially for relapsing and chemo-resistant patients.

*Marsdenia tenacissima* Caulis is a traditional herbal medicine widely grown in the southern provinces (mainly in Yunnan) of China. It is dried from the stems of the Asclepiadaceous plant *Marsdenia tenacissima* (Roxb.) Wight et Arn, and has long been used for treating cancer, asthma, trachitis, tonsillitis, pharyngitis, cystitis, pneumonia and rheumatism in China [5–7]. Promisingly, a water extract of *Marsdenia tenacissimae* [also called Xiao-Ai-Ping (XAP) injection] has been approved to treat cancers in the Chinese market for decades [5]. Clinical studies have shown that administration with *Marsdenia tenacissima* or MTE alone was effective against several cancers, especially for gastric cancer, esophageal cancer, lung cancer, and hepatocellular carcinoma [7–10]. Mechanism studies have demonstrated that MTE or its functional components can inhibit the proliferation and promote apoptosis in human esophageal carcinoma cells [7], non-small cell lung cancer cells [9] and Burkitt's lymphoma cells [11]. However, the potential effectiveness of MTE in leukemia has not yet been fully understood, and the related molecular mechanism is still unknown.

The aim of the present study was to demonstrate the potential roles and molecular mechanisms of MTE in acute T cell leukemia. To this end, we evaluated MTE function in Jurkat cells (T-ALL lines) and lymphocytes from T-ALL patients.We found that MTE strongly inhibited the proliferation and promoted apoptosis in Jurkat cells and lymphocytes from T-ALL patients. Further mechanical studies suggest that PTEN/PI3K/AKT/mTOR signaling pathway mediated the inhibition of cell proliferation by MTE and MTE-induced apoptosis in Jurkat cells. Overall, our results revealed the potent effects of MTE on leukemia therapy and provided experimental evidences in the detailed mechanisms.

## RESULTS

### MTE reduced the viability of T-ALL cell lines

**Figure 1:**
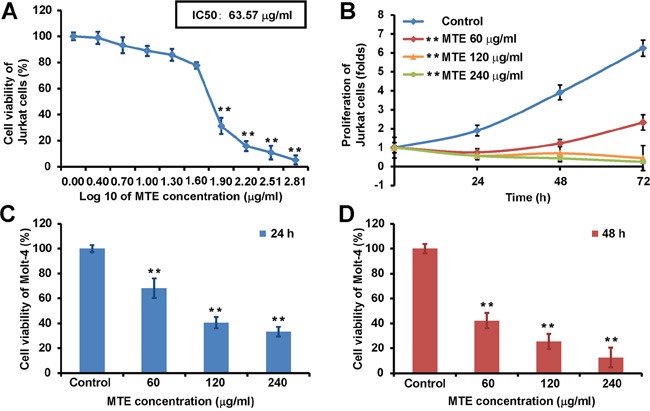
MTE reduced the viability of Jurkat and Molt-4 cell lines **A.** CCK8 assays were performed on Jurkat cells after 24 h of MTE treatment at an ascending concentration range (from 0 to 640 μg/ml) (n=18). Effects on cell viability were presented as a function of μg drug concentration (log scale). Corresponding IC_50_ value was calculated with the appropriate software (graphpad prism). **B.** CCK8 assays were performed on Jurkat cells after 24 h, 48 h, 72 h of MTE treatment at 60, 120, 240 μg/ml, respectively (n=18)(^**^P < 0.01). **C.** CCK8 assays were performed on Molt-4 cells after 24 h of MTE treatment at 60, 120, 240 μg/ml (n=18). **D.** CCK8 assays were performed on Molt-4 cells after 48 h of MTE treatment at 60, 120, 240 μg/ml (n=18). Data were mean ± s.d. ^**^P < 0.01, Student's t-test, compared with control. Controls were treated with 0.1% DMSO.

To examine whether MTE could affect the growth of T-ALL cells, we first performed CCK8 assays by using Jurkat cell lines (T-cell acute lymphoblastic leukemia). Cultured Jurkat cells were treated with different concentrations of MTE from 0 to 640 μg/ml for 24 h, and then cell viability was measured by using CCK8 assays. As shown in Figure 1A, MTE could significantly reduce cell viability of Jurkat cells in a dose-dependent manner. The IC50 values of MTE for Jurkat cells was 63.57 μg/ml (Figure 1A). MTE also could significantly inhibit the growth of Jurkat cells in a time-dependent manner (for 24 h, 48 h and 72 h, p<0.01) (Figure 1B). To further confirm the inhibition of MTE in leukemia cells, we next used another leukemia cell lines, Molt-4 (human acute T lymphoblastic leukemia). Consistently, MTE also could significantly inhibit the growth of Molt-4 after 24h incubation (Figure 1C) and 48h incubation in a dose-dependent manner (Figure 1D). Taken together, these results suggest that MTE reduced the viability of T-ALL cell lines.

### MTE suppressed the proliferation of Jurkat cells by arresting cell cycle at S phase

**Figure 2:**
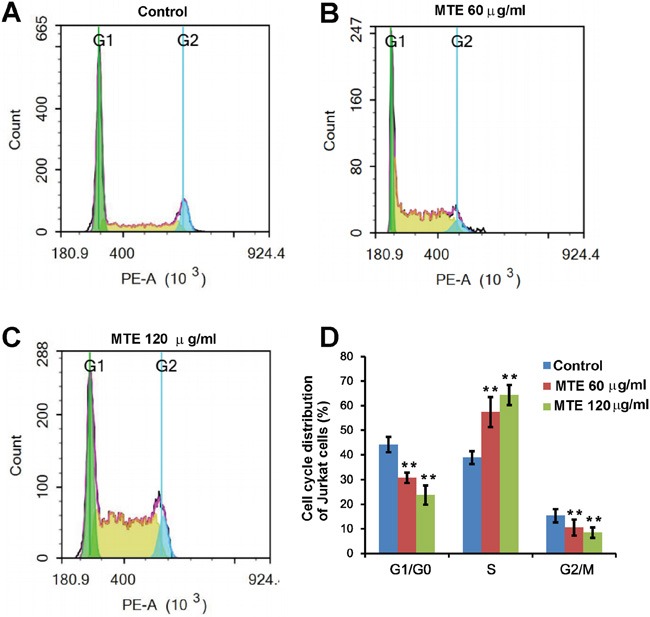
MTE inhibited the proliferation of Jurkat cells by arresting cell cycle at S phase **A-C.** Flow cytometric analysis of cell cycle distributions of Jurkat cells treated by control (A), 60 μg/ml MTE (B), 120 μg/ml MTE treatment (C). **D.** Quantified data of cell cycle distribution of Jurkat cells under various conditions (n=3). Data were mean ± s.d. **P < 0.01, Student's t-test, compared with control. Controls were treated with 0.1% DMSO.

To further determine whether decreased viability of leukemia cells treated by MTE was due to the decrease of cell proliferation, we next examined the effects of MTE on cell cycle distribution in Jurkat cells. As shown in Figure 2A–2D, compared with the control group, the proportion of Jurkat cell in the S phase was significantly increased from 38.92 ± 3.16% to 57.45 ± 3.86% and 64.29 ± 2.18% when cells were treated with 60 μg/ml and 120 μg/ml MTE, respectively. Meanwhile, the percentage of G2/M phase cells was significantly decreased from 15.37 ± 2.68% to 10.54 ± 2.13% and 8.48 ± 3.22%, respectively (Figure 2D). These results suggest that MTE suppressed the proliferation of Jurkat cells by arresting cell cycle at S phase.

### MTE promoted the apoptosis of Jurkat cells

**Figure 3:**
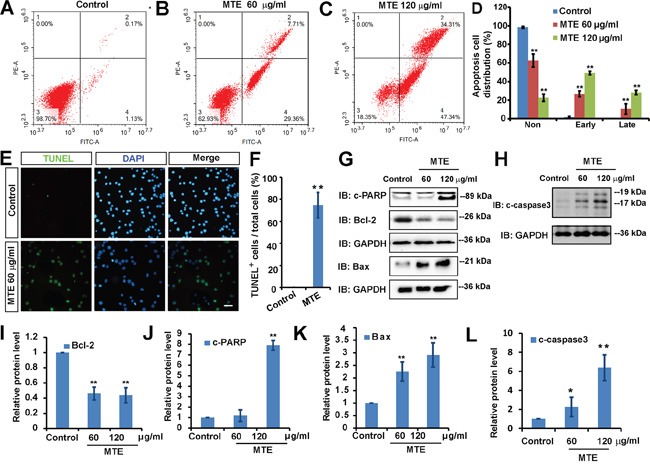
MTE induced the apoptosis of Jurkat cells **A-C.** Flow cytometric analysis of Annexin-V-FITC/PI stained Jurkat cells treated with control (0.1% DMSO, A), MTE 60 μg/ml (B), MTE 120 μg/ml (C). Cells are characterized as healthy cells (bottom left quadrant), necrotic (top left quadrant), early apoptotic (bottom right quadrant), and late apoptotic (top right quadrant). **D.** Quantitative analysis of apoptosis rate of Jurkat cells under various concentrations of MTE as shown in (A-C) (n=3). **E.** TUNEL assay detected the apoptosis of Jurkat cells treated without or with MTE 60 μg/ml for 24 h. Scale bar, 20 μm. **F.** Quantitative analysis of the percentages of TUNEL positive cells over total cells in one filed shown in (E) (n=3). **G** and **H.** Western blot detected the downstream signaling, cleaved-PARP (c-PARP), Bcl-2, Bax (G) and cleaved-Caspase3 (c-Caspase3) (H) by MTE treatment. **I-L.** Quantitative analysis of the relative Bcl-2 (I), c-PARP (J), Bax (K) and c-Caspase3 (L) in (G-H) (n=3 per group, normalized to control). Data were mean ± s.d. **P < 0.01, Student's t-test, compared with control. Controls were treated with 0.1% DMSO.

To further examine whether MTE affect the cell survival of Jurkat cells, we first performed the flow cytometry assays. Jurkat cells were treated with 60 μg/ml or 120 μg/ml MTE for 24 h, and then cells were stained with Annexin-V and propidium iodide (PI) and analyzed by flow cytometry assays. As shown in Figure 3A–3D, compared with control cells, 60 μg/ml or 120 μg/ml MTE treatment significantly increased the apoptosis of Jurkat cells. To further confirm the increase of apoptosis in Jurkat cells by MTE, TUNEL staining assays were performed. As shown in Figure 3E–3F, TUNEL positive cells were significantly increased after the treatment of 60 μg/ml MTE, compared with control. Moreover, western blot results showed that protein level of Bcl-2 (anti-apoptosis protein) in Jurkat cells was significantly decreased after MTE treatment (Figure 3G, 3I), whereas, pro-apoptosis protein, Bax, c-PARP and c-Caspase-3 protein were significantly increased after MTE treatment (Figure 3G–3H, Figure 3J–3L), compared with control group. Taken together, these results strongly suggest that MTE promoted the apoptosis of Jurkat cells in a Caspase-dependent manner.

### MTE enhanced PTEN and inactivated PI3K/AKT/mTOR signaling pathway in Jurkat cells

**Figure 4:**
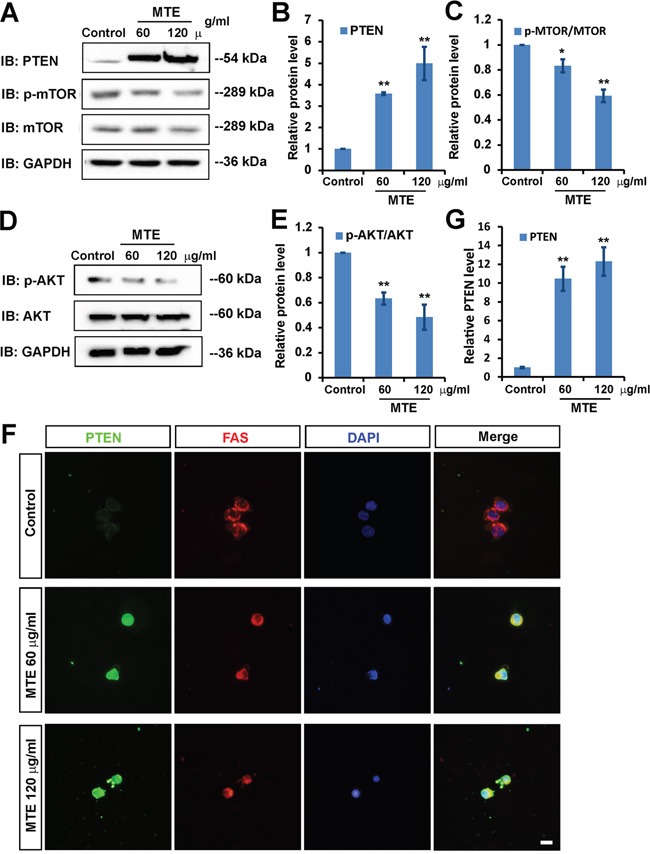
MTE inactivated PI3K/AKT/mTOR signaling pathway though enhancing PTEN **A, D.** Western blot detected the downstream signaling, PTEN, p-mTOR, mTOR (A), and p-AKT, AKT (D) in Jurkat cells by MTE treatment at the indicated concentration for 24 h. **B, C, E.** Quantitative analysis of the relative PTEN (B), p-mTOR/mTOR (C) and p-AKT/AKT (E), as shown in (A, D) (n=3 per group, normalized to control). **F.** Double immunocytochemistry of PTEN (green) and FAS (red) in Jurkat cells treated with MTE at the indicated concentrations for 24 h. Scale bar, 10 μm. **G.** Quantitative analysis of the relative fluorescent PTEN level as shown in (F) (n=20 cells per group, normalized to control). Data were mean ± s.d. *P < 0.05 and **P < 0.01, Student's t-test, compared with control. Controls were treated with 0.1% DMSO.

How does MTE promote the apoptosis of Jurkat cells? Since several studies have reported that phosphatases and tensin homolog (PTEN) is an essential tumor suppressor gene which encodes a phosphatase protein that antagonizes the PI3K/AKT/mTOR anti-apoptotic pathway [12, 13], we next examined whether MTE promoted the apoptosis of Jurkat cells through PTEN pathways. Interestingly, as shown in Figure 4A and 4B, western blot results showed that PTEN protein level was significantly increased by MTE treatment in Jurkat cells, whereas, p-AKT/AKT and p-mTOR/mTOR level were significantly decreased by MTE treatment (Figure 4A, 4C, 4D, 4E). Immunostaining results further confirmed the increase of PTEN in Jurkat cells treated by MTE (Figure 4F and 4G). Taken together, these results suggest that MTE enhanced PTEN and inactivated PI3K/AKT/mTOR signaling pathway in Jurkat cells, which may mediate MTE-induced apoptosis.

### PTEN/PI3K/AKT/mTOR signaling pathway mediated the inhibition of cell proliferation by MTE and MTE-induced apoptosis in Jurkat cells

**Figure 5:**
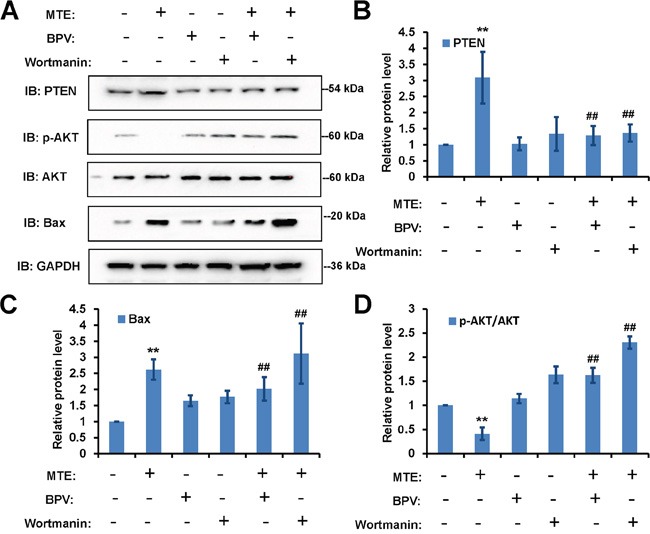
PTEN inhibitor BPV blocked MTE's effects in Jurkat cells, whereas PI3K inhibitor wortmanin enhanced MTE's effects in Jurkat cells **A.** Western blot detected the downstream signaling, PTEN, p-mTOR, mTOR, p-AKT, AKT and Bax in Jurkat cells treated with control, MTE (60 μg/ml) and/or BPV (1 μM) or wortmanin (50 nM). GAPDH as an internal control. **B-D.** Quantitative analysis of the relative PTEN (B), Bax (C), p-AKT/AKT (D) as shown in (A) (n=3 per group, normalized to control). Data were mean ± s.d. ^**^P < 0.01, Student's t-test, compared with control. ^#*#*^*P < 0.01,* Student's t-test, compared with MTE treatment. Controls were treated with 0.1% DMSO.

**Figure 6:**
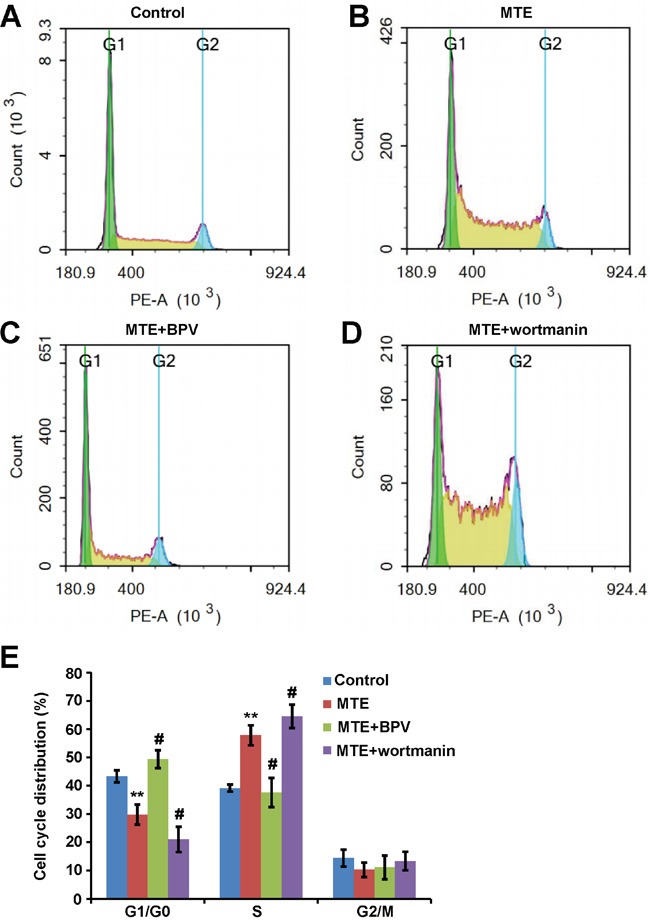
PTEN inhibitor BPV blocked MTE's cell cycle arresting effects, whereas PI3K inhibitor wortmanin enhanced MTE's cell cycle arresting effects in Jurkat cells **A-D.** Representative photographs of cell cycle distributions analyzed by flow cytometer assay. Jurkat cells were treated with control media (A) or MTE (B, 60 μg/ml) or MTE plus BPV (C, 1 μM) or MTE plus wortmanin (D, 50 nM) for 24 h. **E.** Quantified data of cell cycle distribution as shown in (A-D) (n=3). Data were mean ± s.d. ^**^P < 0.01, Student's t-test, compared with control. *^#^P < 0.01* Student's t-test, compared with MTE treatment. Controls were treated with 0.1% DMSO.

**Figure 7:**
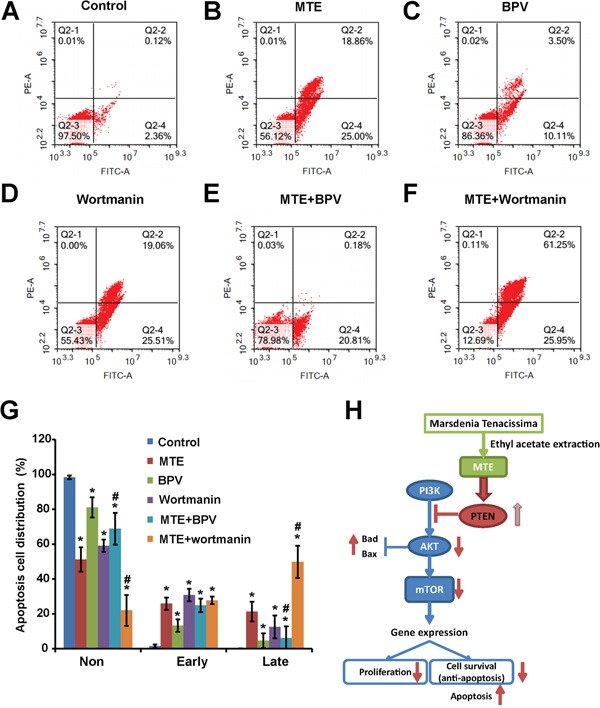
PTEN inhibitor BPV blocked MTE's apoptosis induction effects, whereas PI3K inhibitor wortmanin enhanced MTE's apoptosis induction effects in Jurkat cells **A-F.** Flow cytometric analysis of Annexin-V-FITC/PI stained Jurkat cells treated with control media (A) or MTE (B) or BPV (C) or wortmanin (D) or MTE plus BPV (E) or MTE plus wortmanin (F). Cells are characterized as healthy cells (bottom left quadrant), necrotic (top left quadrant), early apoptotic (bottom right quadrant), and late apoptotic (top right quadrant). **G.** Quantified data of apoptosis rate of Jurkat cells were shown as in (A-F). Data were mean ± s.d. **H.** Working model of signaling pathways induced by MTE and the effects of MTE in T-ALL cells. ^**^P < 0.01, Student's t-test, compared with control. *^#^P < 0.01* Student's t-test, compared with MTE treatment. Controls were treated with 0.1% DMSO.

To further confirm the detail roles of PTEN/PI3K/AKT/mTOR signaling pathway in MTE-induced effect on Jurkat cells, we used the specific drugs to inhibit this pathways. As shown in Figure 5A, western blots indeed showed that BPV (a specific inhibitor of PTEN) could block the increase of PTEN and Bax in Jurkat cells treated by MTE (Figure 5A, 5B, 5C), and also block the decrease of p-AKT/AKT in Jurkat cells treated by MTE (Figure 5A, 5C). By contrast, treatment of MTE with wortmanin, an inhibitor of PI3 kinase, increased p-AKT/AKT and Bax level in Jurkat cells (Figure 5A, 5C, 5D). Moreover, BPV also blocked MTE-induced S arrest in Jurkat cell cycles, whereas wortmanin enhanced MTE-induced S arrest in Jurkat cell cycles (Figure 6). Finally, BPV blocked MTE-induced apoptosis in Jurkat cells, whereas wortmanin enhanced MTE-induced apoptosis in Jurkat cells (Figure 7). Note that wortmanin alone treatment promoted the apoptosis of Jurkat cells (Figure 7D, 7G). Taken together, these results further provide the additional evidence to support that PTEN/PI3K/AKT/mTOR signaling pathway mediates MTE-induced effects (inhibition of cell proliferation and promotion of apoptosis) on Jurkat cells.

### MTE inhibited the proliferation and promoted the apoptosis in T-cell acute lymphoblastic leukemia (T-ALL) cells from patients

**Figure 8:**
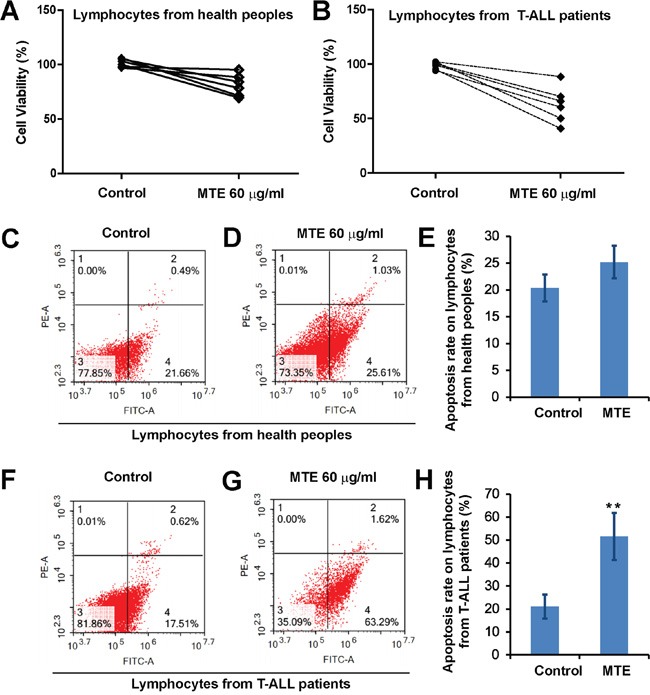
MTE reduced the viability and induced apoptosis of primary lymphocytes from T-ALL patients **A** and **B.** CCK8 assays were performed on lymphocytes from healthy people (A) (n=6) or T-ALL patients (B) (n=6) treated by MTE for 24 h. **C** and **D.** Flow cytometric analysis of Annexin-V-FITC/PI stained lymphocytes from healthy people treated by control (C) or MTE (D) for 24 h. **E.** Quantitative analysis of apoptosis rate of lymphocytes from healthy people as shown in (C-D) (n=6 each group). **F** and **G.** Flow cytometric analysis of Annexin-V-FITC/PI stained lymphocytes from T-ALL patients treated by control (F) or MTE (G) for 24 h. **H.** Quantitative analysis of apoptosis rate of lymphocytes from T-ALL patients as shown in (F-G) (n=6 each group). Data were mean ± s.d. ^**^P < 0.01, Student's t-test, compared with control. Controls were treated with 0.1% DMSO.

To examine whether MTE has the similar effect on T-ALL cells, primary peripheral lymphocytes from 6 different T-ALL patients were collected and treated with MTE. As shown in Figure 8A and 8B, similar to the T-ALL cell lines, MTE also significantly reduced the cell viability of T-ALL patient's lymphocytes, compared with the healthy people's lymphocytes. Moreover, MTE also significantly promoted the apoptosis of T-ALL patient's lymphocytes (Figure 8F, 8G, 8H), compared with the healthy ones (Figure 8C, 8D, 8E). Taken together, these results suggest that MTE inhibited the proliferation and promoted the apoptosis in T-ALL patient's lymphocytes, which implies that MTE is a very potential drug for treatment T-ALL patients.

## DISCUSSION

In this study, we present evidence for MTE's function in the proliferation and apoptosis of T-ALL cells and propose a working model depicted in Figure 7H. In this model, MTE treatment activates PTEN signaling, which inhibits PI3K/AKT/mTOR signaling, antagonizes the PI3K/AKT/mTOR anti-apoptotic pathway, meanwhile inhibits the cell proliferation in T-ALL cells. This study thus not only identifies MTE's unrecognized function in anti-T-ALL cells, but also reveals a novel pathway PTEN/PI3K/AKT/mTOR for the effects of MTE on leukemia therapy.

In China, Xiao-Ai-Ping (XAP) injection is an already Food and Drug Administration (FDA)-approved drug [8]. This drug has anti-tumor effects partly through decreasing the proliferation and increasing the apoptosis of tumor cells such as non-small cell lung cancer cells [14]. Although excellent progress in treatment of T-ALL has been obtained in pediatric patients, an efficient cure is still challenging [4]. Innovative approaches that are more effective and less toxicity are needed. For these reasons, in the present studies, we tested the effects of MTE on the T-ALL cells. Previous studies have shown that under 24 h treatment with the same MTE as we used, MTE inhibited the proliferation of non-small cell lung cancer cell line [9], human esophageal carcinoma cell lines KYSE150 [7]. Consistent with these studies, our results also showed that MTE dose-dependently suppressed the proliferation of T-ALL cells (Figure 1). Furthermore, we also found that significant increase of cell number at S phase and significant decrease at G2/M phase (Figure 2), which suggest that MTE inhibited the proliferation of Jurkat cells by arresting cell cycle at S phase.

Previous studies have shown that MTE promoted the apoptosis of MG63 cells [15], and increased the sensitivity of gefitinib-induced apoptosis of non-small cell lung cancer cells [9]. Consistent with these studies, we also found that MTE significantly induced the apoptosis of Jurkat cells based on Annexin V-FITC/PI-stained flow cytometry and TUNEL staining assays (Figure 3). Bax is a pro-apoptotic protein, whereas Bcl-2 is an essential anti-apoptotic protein. It is a well-known fact that the decrease of Bcl-2 enables the oligomerized Bax to re-localize and insert into outer mitochondrial membrane drive apoptosis [16]. Interestingly, we found that MTE treatment decreased the expression of Bcl-2 and increased Bax level in Jurkat cells, which suggested that the enhanced apoptosis of Jurkat cells by MTE treatment may be due to the activation of mitochondrial apoptotic pathways.

How did MTE decrease Bcl-2 level and increase Bax level? Recent studies have shown that the dysfunctions of PTEN/PIK3/AKT/mTOR pathways are involved in tumorigenesis [13, 17]. In this signaling cascade, PTEN is an essential tumor suppressor gene, and plays a central negative regulator through removing the D3 phosphate on the inositol ring of PIP3, counteracting PI3K and down-regulating the PI3K/AKT/mTOR signaling pathway which affects cell growth, proliferation and survival [18]. Impairment of this tumor suppressor pathway potentially became a causal factor for development of malignancies. Interestingly, mutant Notch1 can activate c-Myc and PI3K/AKT/mTOR signaling in T-ALL [19–21]. PI3K inhibitors impair the proliferation and survival of T-ALL cells [22, 23]. PTEN is involved in regulating downstream effects of Noth1 signaling such as proliferation and survival of T-cell progenitors [24]. Loss of function of PTEN mutations leading to constitutive activation of Akt were identified in T-ALL cell lines [25]. Interestingly, several studies have now described PTEN loss through mutation and/or genomic deletion in up to 35% of pediatric patients with T-ALL [26–32]. Based on above evidence, in this study, we tested whether MTE induced apoptosis and inhibited proliferation of Jurkat cells through antagonizing the PI3K/AKT/mTOR anti-apoptotic pathway via enhancement of PTEN. We found that MTE treatment increased PTEN level and decreased p-AKT/AKT and p-mTOR/m-TOR (Figure 4). Furthermore, PTEN inhibitor BPV blocked MTE-induced apoptosis and the inhibition of proliferation of Jurkat cells treated by MTE, whereas PI3 kinase inhibitor wortmanin enhanced the MTE-induced apoptosis and the inhibition of proliferation of Jurkat cells treated by MTE (Figure 5, Figure 6 and Figure 7). These results strongly imply that PTEN/PI3K/AKT/mTOR signaling pathways mediates the effects of MTE on Jurkat cells, and provide a novel tool for treatment T-ALL, enhancement PTEN levels.

In our study, most of experiments were performed in Jurkat cell lines. To test whether MTE has the similar effect on T-ALL cells, primary peripheral lymphocytes from 6 different T-ALL patients were collected and treated with MTE. We found that MTE also could inhibit the proliferation and induce the apoptosis of primary peripheral lymphocytes from T-ALL patients. These results imply that MTE has a very promising clinical application to treatment T-ALL patients. Thus, it is very interest to test the idea that MTE as a novel drug to treat T-ALL patients in future. Meanwhile, MTE is a complex drug, which has many components. So, identification of which component has strongest effects on T-ALL cells needs further investigation.

In summary, the present study demonstrated that MTE could inhibit the proliferation and induce the apoptosis of Jurkat cells and T-ALL cells, which mediated by PTEN/PI3K/AKT/mTOR signaling pathways.Our results revealed the novel effects of MTE on leukemia therapy and provided experimental evidences on the detailed mechanism.

## MATERIALS AND METHODS

### Reagents

*Marsdenia tenacissimae* Extracts (MTE) trade name: Xiao-Ai-Ping injection; 1 g crude/ml was purchased from SanHome Pharmaceutical Co., Ltd. (Nanjing, China). The stem of *Marsdenia tenacissimae* was harvested from Yunnan, China and the preparation of MTE was as follows [33]: 1 kg powder of *Marsdenia tenacissimae* stem was extracted by ethyl acetate for three times. The extracts were filtered and concentrated, followed by re-suspended with 85% (v/v) Ethyl acetate and centrifuged at 4°C for three times. The final suspension was evaporated to approximately 200 ml. MTE was dissolved in dimethyl sulfoxide (DMSO, Sigma-Aldrich, St. Louis, MO, USA).

Potassium bisperoxo (1, 10-phenanthroline) oxovanadate (bpV[phen]), (Sigma-Aldrich, St. Louis, MO, USA), PTEN inhibitor, were used at 1 μM in DMSO. Wortmanin (Sigma-Aldrich, St. Louis, MO, USA), a specific PI3K inhibitor, was used at 50 nM in DMSO.

### Cell culture and primary samples

The leukemia cell lines Jurkat (T-cell acute lymphoblastic leukemia), MOLT-4 (human acute T lymphoblastic leukaemia) were grown in RPMI 1640 supplemented with 10% heat-inactivated fetal bovine serum (FBS), and were exposed to MTE as indicated concentrations.

Samples from T-ALL patients were obtained with informed consent according to Institutional guidelines. Lymphocytes were isolated though using Lymphocyte Separation Medium (LSM) (TBD, Tianjin, China). Briefly, 6 newly diagnosed T-ALL patients from Zhejiang Provincial People's Hospital (Jan, 2016, Apr, 2016) and 6 healthy patients were recruited with informed consent. 5 mL anti-coagulated blood was collected from each people. Anti-coagulated blood was diluted 1:1 with calcium-magnesium-free PBS, and was layered onto 6 mL LSM. After centrifuged 400 g for 20 min, interface layer was collected and diluted with 20 mL RPMI 1640, then were centrifuged 70 g for another 10 min. Pellet was re-suspended and grown in RPMI 1640 supplemented with 20% FBS and ITS (insulin-transferrin-sodium selenite), and followed by MTE treatment. Control refers to cells were treated with 0.1% DMSO.

### Cell viability assay

Cell viability was assessed using CCK8 colorimetric assay (Beyotime, Hangzhou, China) as previously described [34]. Briefly, cells were plated in 96-well plates (2 × 10^3^cells/well) at 37 with 5% CO2 for 24 h, 48 h and 72 h, and the absorbance was recorded at 450 nm using a micro-plate reader (BIOTEK, Vermont, USA). The results are expressed as viability rates. Cell viability was calculated according to the following formula: inhibition rate=1-(OD treatment-OD blank)/(OD treatment-OD blank).

### Cell cycle assay

Cell cycle was measured by using the Cell Cycle Staining Kit (MULTI SCIENCES, Hangzhou, China) following the manufacturer's instructions after drug treatment. Briefly, cells were re-suspended and mixed in DNA staining solution. After incubation for 30 min, cell cycle was detected with a flow cytometry (ACEA NovoCyte, USA). The results are presented as percentage of cells at each phase of cell cycle.

### Apoptosis assay

Cell apoptosis was measured by using the Annexin V-FITC/PI Apoptosis Detection Kit (Beyotime, Haimen, China) following the manufacturer's instructions after drug treatment. Briefly, cells were re-suspended and mixed in 500 μl binding buffer containing 5 μl Annexin V-FITC and 5 μl PI. After incubation for 15 min, cell apoptosis was detected with a flow cytometry (ACEA NovoCyte, USA).

### Western blot

Cells were lysed with RIPA lysate (Beyotime, Haimen, China) to extract the total proteins. Then the concentration of total proteins was quantified by a bicinchoninic acid (BCA) protein assay kit (Beyotime). Equal amounts of proteins were loaded and separated by SDS-polyacrylamide gel electrophoresis (PAGE) followed by transferred onto polyvinylidene fluoride (PVDF) membranes (Millipore, Bedford, MA, USA). After being blocked with 5% non-fat milk for 1 h, the blots were probed with primary antibodies against p-mTOR(Ser2448) (1:1000, Cell Signaling Technology, USA), mTOR (1:1000, Cell Signaling Technology), Bax (1:1000, Cell Signaling Technology), Bcl-2 (1:1000, Cell Signaling Technology), cleaved-PARP (1:1000, Cell Signaling Technology), PTEN (1:1000, Cell Signaling Technology), p-AKT(Ser473) (1:1000, Cell Signaling Technology), AKT (1:1000, Cell Signaling Technology), cleaved-Caspase-3 (1:1000, Cell Signaling Technology), GAPDH (1:1000, Cell Signaling Technology, USA) at 4°C overnight and incubated subsequently with their corresponding secondary antibodies (1:5000, Beyotime) for 1 h. An enhanced chemiluminescence (ECL) solution (Qihai Biotec, Shanghai, China) was used to visualize the target bands, and the Gel-Pro-Analyzer software (Bethesda, MD, USA) was employed to measure relative band intensities. GAPDH was served as an internal control.

### Immunocytochemistry

Jurkat cells were plated on poly-L-lysine-coated glass coverslips. After treatment, cells were fixed with fresh 4% PFA in 0.1 M PBS (pH 7.4) for 20 min. After washing with PBS, cells were permeabilized with 0.1% Triton X-100 in 0.1 M PBS for 5 min, followed by incubation in blocking buffer (5% BSA and 0.1% Triton X-100 in 0.1 M PBS, pH 7.4) for 1 h, and incubated overnight at 4°C with primary antibodies diluted in the blocking buffer. Cells were washed 3 times with PBS and incubated for 1 h at room temperature with an appropriate fluorescence-conjugated secondary antibody (1:1000, Molecular probes). The primary antibodies were rabbit polyclonal antibodies against PTEN (1:500, CST), or with a monoclonal antibodies against FAS (1:200, Abcam). Cells were stained for DAPI (1:1000, Molecular Probes) to visualize nucleus. Images were acquired on a Fluorescence microscope (Olympus).

### Statistics

Data were expressed as means ± standard deviation (SD). All data presented represent results from at least 3 independent experiments. Statistical analysis was performed using Student's t-test, or using an ANOVA with pair-wise comparisons. Statistical significance was defined as P < 0.05.
